# Supplementary material for: Polyphasic discrimination of Shewanella seohaensis from closely related species and a whole-genome multilocus (wgMLST) scheme for the evaluation of diversity within this Shewanella clade
Source: Appl Environ Microbiol. 2025 Aug 13;91(9):e01189-25. doi: 10.1128/aem.01189-25 (PMC12442382; doi:10.1128/aem.01189-25)
Supplement: Supplemental tables — Tables S1 to S4. [file aem.01189-25-s0001.pdf]

Polyphasic discrimination of *Shewanella seohaensis* from closely related species and a whole-genome multilocus (wgMLST) scheme for the evaluation of diversity within this *Shewanella* clade

Maria de Oliveira Firmino [a,b,1], Mykyta Forofontov [b,1], Ricardo Soares [a,c], Ricardo O. Louro [a], Alberto J. Martín-Rodríguez [d,e], Mário Ramirez [b], Catarina M. Paquete [a,#]

Supplementary material

Supplementary Table 1. List of all the assembly identifiers used in Whole-genome Multilocus Sequence Typing (wgMLST) scheme creation. The last 11 entries, in bold, correspond to the were excluded from the final analysis due to quality concerns (e.g., incomplete assemblies, contamination, or low sequencing depth).

Species	Strain	Assembly Name	Assembly Accession	BioSample
S. baltica	OS155	ASM1584v1	GCA_000015845.1	SAMN02598299
S. baltica	OS185	ASM1732v1	GCA_000017325.1	SAMN02598398
S. baltica	OS195	ASM1876v1	GCA_000018765.1	SAMN00623061
S. baltica	OS223	ASM2166v1	GCA_000021665.1	SAMN00623062
S. oneidensis	MR-1	ASM14616v2	GCA_000146165.2	SAMN02604014
S. baltica	BA175	ASM14773v3	GCA_000147735.3	SAMN00016994
S. baltica	OS678	ASM17887v2	GCA_000178875.2	SAMN00016780
S. baltica	OS183	ASM17953v2	GCA_000179535.2	SAMN02256524
S. baltica	OS117	ASM21589v1	GCA_000215895.1	SAMN00713637
S. baltica	OS625	ASM23134v2	GCA_000231345.2	SAMN02256537
S. decolorationis	S12	SheDec2.0	GCA_000485795.1	SAMN02469853
S. putrefaciens	JCM 20190	ASM61500v1	GCA_000615005.1	SAMD00004020
S. xiamenensis	BC01	SXM1.0	GCA_000712635.2	SAMN02678192
S. putrefaciens	NBRC 3908	ASM159132v1	GCA_001591325.1	SAMD00046716
S. baltica	M1	ASM162032v1	GCA_001620325.1	SAMN04555237
S. xiamenensis	T17	ASM172319v1	GCA_001723195.1	SAMN03704056
S. xiamenensis	T17	ASM207485v1	GCA_002074855.1	SAMN03397547
S. putrefaciens	SA70	ASM215736v2	GCA_002157365.2	SAMN06673971

S. bicestrii	JAB-1	ASM221687v1	GCA_002216875.1	SAMN07326684
S. xiamenensis	AS58	ASM273801v1	GCA_002738015.1	SAMN07830263
S. morhuae	CW7	ASM302829v1	GCA_003028295.1	SAMN08793905
S. baltica	CW2	ASM303092v1	GCA_003030925.1	SAMN08768697
S. putrefaciens	WS13	ASM304425v1	GCA_003044255.1	SAMN08180388
S. baltica	128	ASM305276v1	GCA_003052765.1	SAMN08730657
S. xiamenensis	DCB2-1	ASM313054v1	GCA_003130545.1	SAMN09197335
S. oneidensis	S2_009_000_R2_72	ASM324122v1	GCA_003241225.1	SAMN07426572
S. putrefaciens	97	ASM331542v1	GCA_003315425.1	SAMN09062819
S. decolorationis	Sesselensis	ASM435430v1	GCA_004354305.1	SAMN10916473
S. xiamenensis	ZYW2	ASM759522v1	GCA_007595225.1	SAMN04492228
S. xiamenensis	ZYW3	ASM759526v1	GCA_007595265.1	SAMN04492229
S. xiamenensis	ZYW4	ASM759527v1	GCA_007595275.1	SAMN04492230
S. xiamenensis	ZYW6	ASM759528v1	GCA_007595285.1	SAMN04492232
S. xiamenensis	ZYW5	ASM759533v1	GCA_007595335.1	SAMN04492231
S. xiamenensis	ZYW1	ASM763646v1	GCA_007636465.1	SAMN04492227
S. decolorationis	Ni1-3	ASM792304v4	GCA_007923045.4	SAMN09837225
S. putrefaciens	FDAARGOS_681	ASM973057v1	GCA_009730575.1	SAMN11056396
S. xiamenensis	SxND_W2_2018	ASM1249034v1	GCA_012490345.1	SAMD00215989
S. xiamenensis	SxND_W5_2018	ASM1249046v1	GCA_012490465.1	SAMD00215990
S. xiamenensis	SxND_W9_2018	ASM1249060v1	GCA_012490605.1	SAMD00215991
S. oncorhynchi	S-1	ASM1258445v1	GCA_012584455.1	SAMN14596183
S. oneidensis	MR-1	ASM1326721v1	GCA_013267215.1	SAMN14968595
S. xiamenensis	JCM 16212	ASM1464713v1	GCA_014647135.1	SAMD00245260
S. putrefaciens	JCM 20190	ASM1464755v1	GCA_014647555.1	SAMD00245284
S. putrefaciens	CGMCC-1.6515	ASM1640630v1	GCA_016406305.1	SAMN17120582
S. putrefaciens	ATCC 8071	ASM1640632v1	GCA_016406325.1	SAMN17120581
S. putrefaciens	XY07	ASM1706819v1	GCA_017068195.1	SAMN17981346
S. xiamenensis	111D	ASM1926612v1	GCA_019266125.1	SAMN06461335
S. xiamenensis	111B	ASM1926620v1	GCA_019266205.1	SAMN06461331
S. acanthi	FJAT-51860	ASM1945747v1	GCA_019457475.1	SAMN20446335
S. mangrovisoli	FJAT-51754	ASM1945763v1	GCA_019457635.1	SAMN20446334
S. putrefaciens	YZ08	ASM1959908v1	GCA_019599085.1	SAMN20607798
S. putrefaciens	YZ-J	ASM1959912v1	GCA_019599125.1	SAMN20608154
S. glacialipiscicola	T147	ASM1965523v1	GCA_019655235.1	SAMD00284632
S. hafniensis	ATCC BAA-1207	ASM1965533v1	GCA_019655335.1	SAMD00284633
S. morhuae	ATCC BAA-1206	ASM1965537v1	GCA_019655375.1	SAMD00284634
S. xiamenensis	NUITM-VS1	ASM1997365v1	GCA_019973655.1	SAMD00396922
S. putrefaciens	M101	ASM2021669v1	GCA_020216695.1	SAMN16861155
S. xiamenensis	S2C505	ASM2120930v1	GCA_021209305.1	SAMN23440126
S. shenzhenensis	A25	ASM2232155v1	GCA_022321555.1	SAMN25610639
S. xiamenensis	CQ-Y1	ASM2245380v1	GCA_022453805.1	SAMN26156289
S. shenzhenensis	A25	ASM2266227v1	GCA_022662275.1	SAMN25525559

S. glacialipiscicola	LMG 23744	ASM2328348v1	GCA_023283485.1	SAMN24537895
S. hafniensis	KCTC 22180	ASM2328358v1	GCA_023283585.1	SAMN24537889
S. xiamenensis	DSM 22215	ASM2328366v1	GCA_023283665.1	SAMN24537882
S. profunda	DSM 15900	ASM2328376v1	GCA_023283765.1	SAMN24537880
S. xiamenensis	28.1.37	ASM2465572v1	GCA_024655725.1	SAMN29581404
S. baltica	11FHM2	ASM2480360v1	GCA_024803605.1	SAMN14595194
S. xiamenensis	HD6416	ASM2497175v1	GCA_024971755.1	SAMN20179462
S. xiamenensis	HD6452	ASM2497279v1	GCA_024972795.1	SAMN20844899
S. baltica	20FHA1-2	ASM2499887v1	GCA_024998875.1	SAMN13656461
S. baltica	F1MM1	ASM2499888v1	GCA_024998885.1	SAMN13656463
S. baltica	20FHM1	ASM2499892v1	GCA_024998925.1	SAMN13656462
S. baltica	20FHA1	ASM2499894v1	GCA_024998945.1	SAMN13656460
S. baltica	19FHA2-2	ASM2499896v1	GCA_024998965.1	SAMN13656459
S. baltica	18FHM1	ASM2499898v1	GCA_024998985.1	SAMN13656457
S. baltica	19FHA1	ASM2499899v1	GCA_024998995.1	SAMN13656458
S. baltica	16FHM3	ASM2499902v1	GCA_024999025.1	SAMN13656455
S. baltica	15FHM4	ASM2499904v1	GCA_024999045.1	SAMN13656447
S. baltica	16FHA1	ASM2499906v1	GCA_024999065.1	SAMN13656452
S. baltica	15FHM1	ASM2499907v1	GCA_024999075.1	SAMN13656446
S. baltica	17FHM1	ASM2499910v1	GCA_024999105.1	SAMN13656456
S. baltica	15FHA3	ASM2499912v1	GCA_024999125.1	SAMN13656445
S. baltica	15FHA2-2	ASM2499914v1	GCA_024999145.1	SAMN13656444
S. baltica	14FHA1	ASM2499916v1	GCA_024999165.1	SAMN13656436
S. baltica	15FHA1	ASM2499917v1	GCA_024999175.1	SAMN13656443
S. baltica	13FHM1	ASM2499920v1	GCA_024999205.1	SAMN13656434
S. baltica	12FHM1	ASM2499921v1	GCA_024999215.1	SAMN13656428
S. baltica	11FHM1	ASM2499924v1	GCA_024999245.1	SAMN13656419
S. baltica	9FHM1	ASM2499932v1	GCA_024999325.1	SAMN13656412
S. baltica	7FHA1	ASM2499935v1	GCA_024999355.1	SAMN13656409
S. baltica	8FHM1	ASM2499937v1	GCA_024999375.1	SAMN13656411
S. baltica	7FHM1	ASM2499942v1	GCA_024999425.1	SAMN13656410
S. baltica	6FHM1	ASM2499950v1	GCA_024999505.1	SAMN13656407
S. baltica	5FHM1	ASM2499951v1	GCA_024999515.1	SAMN13656404
S. baltica	5FHA2	ASM2499956v1	GCA_024999565.1	SAMN13656403
S. baltica	5FHA1	ASM2499960v1	GCA_024999605.1	SAMN13656402
S. baltica	1FHM1	ASM2499968v1	GCA_024999685.1	SAMN13656391
S. baltica	2FMM1	ASM2499969v1	GCA_024999695.1	SAMN13656399
S. baltica	2FHM1	ASM2499971v1	GCA_024999715.1	SAMN13656395
S. baltica	1FHA1	ASM2499976v1	GCA_024999765.1	SAMN13656389
S. baltica	4FHA1	ASM2499978v1	GCA_024999785.1	SAMN13656401
S. septentrionalis	SP1W3	ASM2537026v1	GCA_025370265.1	SAMN28933880
S. xiamenensis	HD6449	ASM2539747v1	GCA_025397475.1	SAMN20844898
S. xiamenensis	HD6446	ASM2539755v1	GCA_025397555.1	SAMN20844897

S. xiamenensis	HD6443	ASM2539756v1	GCA_025397565.1	SAMN20844896
S. xiamenensis	HD6424	ASM2539761v1	GCA_025397615.1	SAMN20844895
S. xiamenensis	HD6420	ASM2539762v1	GCA_025397625.1	SAMN20844894
S. putrefaciens	DSM 50426	ASM2539793v1	GCA_025397935.1	SAMN27561646
S. putrefaciens	4H	ASM2540287v1	GCA_025402875.1	SAMN30884599
S. glacialipiscicola	SM91	ASM2565360v1	GCA_025653605.1	SAMN25232830
S. glacialipiscicola	SM77	ASM2565389v1	GCA_025653895.1	SAMN25232827
S. baltica	14FHM1	ASM2591965v1	GCA_025919655.1	SAMN13656439
S. xiamenensis	NUITM-VS2	ASM2599831v1	GCA_025998315.1	SAMD00529478
S. xiamenensis	NUITM-VS3	ASM2601257v1	GCA_026012575.1	SAMD00529479
S. xiamenensis	2015oxa	ASM2971942v1	GCA_029719425.1	SAMN11164563
S. xiamenensis	GD03933	ASM2983954v1	GCA_029839545.1	SAMN30525389
S. xiamenensis	GD03733	ASM2984334v1	GCA_029843345.1	SAMN30525189
S. xiamenensis	NF-10	ASM3000836v1	GCA_030008365.1	SAMN31046451
S. xiamenensis	NF-11	ASM3000837v1	GCA_030008375.1	SAMN31046452
S. xiamenensis	NF-9	ASM3000838v1	GCA_030008385.1	SAMN31046450
S. xiamenensis	NF-12	ASM3000845v1	GCA_030008455.1	SAMN31046453
S. xiamenensis	NF-5	ASM3000847v1	GCA_030008475.1	SAMN31046446
S. xiamenensis	NF-8	ASM3000849v1	GCA_030008495.1	SAMN31046449
S. xiamenensis	NF-6	ASM3000850v1	GCA_030008505.1	SAMN31046447
S. xiamenensis	NF-7	ASM3000851v1	GCA_030008515.1	SAMN31046448
S. xiamenensis	NF-4	ASM3000857v1	GCA_030008575.1	SAMN31046445
S. xiamenensis	NF-3	ASM3000859v1	GCA_030008595.1	SAMN31046444
S. xiamenensis	NF-1	ASM3000860v1	GCA_030008605.1	SAMN31046442
S. xiamenensis	NF-2	ASM3000861v1	GCA_030008615.1	SAMN31046443
S. xiamenensis	FH-1	ASM3001254v1	GCA_030012545.1	SAMN31045518
S. decolorationis	NBRC 103170	ASM3016049v1	GCA_030160495.1	SAMD00238702
S. glacialipiscicola	NBRC 102030	ASM3016183v1	GCA_030161835.1	SAMD00582132
S. xiamenensis	NZRM 825	ASM3028104v1	GCA_030281045.1	SAMN28613652
S. oncorhynchi	Z-P2	ASM3084876v1	GCA_030848765.1	SAMN36977568
S. putrefaciens	3469	ASM3145673v1	GCA_031456735.1	SAMN18245696
S. baltica	PB313	ASM3158399v1	GCA_031583995.1	SAMN37271891
S. xiamenensis	RK-E270.1	ASM3303118v1	GCA_033031185.1	SAMN35054493
S. xiamenensis	PP-E493	ASM3303171v1	GCA_033031715.1	SAMN35054492
S. baltica	DSS12	ASM3348487v1	GCA_033484875.1	SAMN37205435
S. oneidensis	ATCC 700550	ASM3384304v1	GCA_033843045.1	SAMN38186254
S. xiamenensis	160P	ASM3632420v1	GCA_036324205.1	SAMN39589607
S. oncorhynchi	B	ASM3632448v1	GCA_036324485.1	SAMN39327834
S. xiamenensis	8M34	ASM3632696v1	GCA_036326965.1	SAMN36378994
S. xiamenensis	8M38	ASM3632698v1	GCA_036326985.1	SAMN36378995
S. oneidensis	MR-1	ASM3632702v1	GCA_036327025.1	SAMN37200116
S. baltica	TMP1	Shew_sp_01_v1	GCA_037094655.1	SAMN40213720
S. mangrovisoli	29	ASM3843239v1	GCA_038432395.1	SAMN40911144

S. xiamenensis	F93	ASM3843240v1	GCA_038432405.1	SAMN40911142
S. xiamenensis	16	ASM3843245v1	GCA_038432455.1	SAMN40911143
S. xiamenensis	F95	ASM3843246v1	GCA_038432465.1	SAMN40911141
S. xiamenensis	F130	ASM3843249v1	GCA_038432495.1	SAMN40911140
S. xiamenensis	F150	ASM3843250v1	GCA_038432505.1	SAMN40911139
S. xiamenensis	F104	ASM3843253v1	GCA_038432535.1	SAMN40911138
S. xiamenensis	F96	ASM3843256v1	GCA_038432565.1	SAMN40911137
S. xiamenensis	F139	ASM3843258v1	GCA_038432585.1	SAMN40911135
S. xiamenensis	F143	ASM3843259v1	GCA_038432595.1	SAMN40911136
S. xiamenensis	F141	ASM3843262v1	GCA_038432625.1	SAMN40911133
S. xiamenensis	F152	ASM3843264v1	GCA_038432645.1	SAMN40911134
S. xiamenensis	F137	ASM3843266v1	GCA_038432665.1	SAMN40911132
S. xiamenensis	F131	ASM3843268v1	GCA_038432685.1	SAMN40911131
S. xiamenensis	H282	ASM3843269v1	GCA_038432695.1	SAMN40911130
S. xiamenensis	H334	ASM3843271v1	GCA_038432715.1	SAMN40911127
S. xiamenensis	H402	ASM3843272v1	GCA_038432725.1	SAMN40911129
S. xiamenensis	H337	ASM3843274v1	GCA_038432745.1	SAMN40911128
S. xiamenensis	H99	ASM3843278v1	GCA_038432785.1	SAMN40911123
S. mangrovisoli	H318	ASM3843279v1	GCA_038432795.1	SAMN40911125
S. xiamenensis	H307	ASM3843280v1	GCA_038432805.1	SAMN40911122
S. xiamenensis	H333	ASM3843281v1	GCA_038432815.1	SAMN40911124
S. xiamenensis	H336	ASM3843283v1	GCA_038432835.1	SAMN40911126
S. xiamenensis	H233	ASM3843289v1	GCA_038432895.1	SAMN40911121
S. xiamenensis	H241	ASM3843292v1	GCA_038432925.1	SAMN40911120
S. baltica	CA	ASM3976233v1	GCA_039762335.1	SAMN41422631
S. baltica	3A	ASM3987150v1	GCA_039871505.1	SAMN41424287
S. morhuae	ATCC BAA-1205	-	GCA_900156405.1	SAMN05421840
S. baltica	NCTC10735	50884_G01	GCA_900456975.1	SAMEA4442456
S. morhuae	NCTC10736	44738_B01	GCA_900457045.1	SAMEA3879481
S. putrefaciens	NCTC10695	57675_D02	GCA_900457065.1	SAMEA104224778
S. putrefaciens	NCTC12093	43781_G01	GCA_900636665.1	SAMEA3724093
S. baltica	NCTC10737	44858_H01	GCA_900636855.1	SAMEA3881057
S. hafniensis	T2.3D-1.1	T2.3D-1.1	GCA_902728295.3	SAMEA6455789
S. oneidensis	SRR9109399	SRR9109399	GCA_945952185.1	SAMEA110422286
S. baltica	SF1039	SF1039	GCA_949794895.1	SAMEA112774578
S. baltica	-	piPlaSpea1	GCA_963676695.1	SAMEA114407665
S. seohaensis	BC20	ASM312958v1	GCF_003129585.1	SAMN07156272
S. seohaensis	CCUG 60900	ASM2328377v1	GCF_023283775.1	SAMN24537878
S. seohaensis	This Study	This Study	This Study	This Study
S. xiamenensis	This Study	This Study	This Study	This Study
S. seohaensis	JAB-1	ASM221687v1	GCA_002216875.1	SAMN07326684
S. seohaensis	NCTC12093	43781_G01	GCA_900636665.1	SAMEA3724093
S. seohaensis	SA70	ASM215736v2	GCA_002157365.2	SAMN06673971

S. seohaensis	GD03713	ASM2984370v1	GCF_029843705.1	SAMN30525169
S. putrefaciens	**SRR6727992**	**CAMSMN01**	**GCA_947060975.1**	**SAMEA111463821**
S. hafniensis	**EEHMALJP1**	**CACVAL02**	**GCA_902726625.2**	**SAMEA6457477**
S. baltica	**ERR2206780**	**CALFPE01**	**GCA_937919145.1**	**SAMEA14074890**
S. xiamenensis	**v8ult0W3t9_bin.4**	**CALUAW01**	**GCA_943914135.1**	**SAMEA110089393**
S. oneidensis	**SRR12456162**	**CAMFLO01**	**GCA_945957515.1**	**SAMEA110424202**
S. putrefaciens	**ERR3519528**	**CALMPT01**	**GCA_937871465.1**	**SAMEA14036488**
S. baltica	**UBA8873**	**ASM351664v1**	**GCA_003516645.1**	**SAMN08019569**
S. putrefaciens	**HRCR-6**	**ASM51906v1**	**GCA_000519065.1**	**SAMN02584939**
S. xiamenensis	**S3C505**	**ASM2120924v1**	**GCA_021209245.1**	**SAMN23440129**
S. bicestrii	**USCH1**	**ASM3761919v1**	**GCA_037619195.1**	**SAMN32961353**
S. seohaensis	**KCTC 23556**	**ASM2544921v1**	**GCF_025449215.1**	**SAMN30945948**

Supplementary Table 2. Phenotypic features of *S. seohaensis* DSM9451, *S. seohaensis* SA70, *S. seohaensis* CCUG60900, *S. xiamenensis* HI32664, S. *putrefaciens* 95 and *S. decolorationis* JCM21555. +: Positive reaction; -: Negative reaction; (+): low intensity but considered positive; (-): low intensity but considered negative; All phenotypic information regarding *S. decolorationis* JCM21555 is from the literature (1, 2). On the other species, the results from the literature are marked with a "*". The shaded cells highlight differences between the results and the literature.

**Strains**		**DSM9451**	**SA70**	**CCUG60900**	**HI32664**	**95**	**JCM21555**	**Notes**
**Growth**	**4 ºC**	-	-	-	-	+	**ND**	**Distinct phenotype for *S. putrefaciens* 95**
	**37 ºC**	+	+	+	+	(+)	+*	
	**40 ºC**	+	+	+	+	-	+*	Regarding the literature, the ***S. xiamenensis* type strain** does not grow at 40ºC
	**42 ºC**	-	-	-	-	-	**ND**	
**KIA Test**	**H_2S Production**	+	+	+	+	+	+*	Regarding the literature, *S. seohaensis* **CCUG60900** does not produce H_2S
	**Fermentation of Glucose**	-	-	-	-	+	**ND**	**Distinct phenotype for *S. putrefaciens* 95**
	**Fermentation of Lactose**	-	-	-	-	-	**ND**	
	**Gas Production**	-	-	-	-	-	**ND**	
**G + C %**		**47.87**	**47.89**	**48.19**	**46.28**	**44.39**	**47.09**	**Distinct phenotype between species**
**API ZYM**	**2 - Alkaline Phosphatases**	+	+	+	+	+	+*	
	**3 - Butyrate Esterase**	+	+	+	+	+	+*	

	**4 - Caprylate Esterase Lipase**	+	+	+	+	+	+*	
	**5 - Lipase (C 14)**	-	-	-	-	-	-*	
	**6 - Leucine Aminopeptidases**	+	+	+	+	+	-*	**Distinct phenotype for *S. decolorationis* JCM21555**
	**7 - Valine Aminopeptidases**	-	-	-	-	-	-*	Regarding the literature, the ***S. xiamenensis* type strain** has valine aminopeptidase activity
	**8 - Cystine Aminopeptidases**	-	-	-	-	-	-*	
	**9 - Trypsin**	+	+	+	(-)	+	+*	Regarding the literature, ***S. seohaensis* CCUG60900** does not have trypsin activity **Distinct phenotype for *S. xiamenensis* HI32664**
	**10 - α-chymotrypsin**	+	+	+	+	+	+*	
	**11 - Acid Phosphatases**	+	+	+	+	+	+*	
	**12 - Naphthol-AS-BI-phosphohydrolase**	**ND**	**ND**	+*	+*	**ND**	+*	
	**13 - α-galactosidase**	-	-	-	-	-	-*	
	**14 - β-galactosidase**	-	-	-	-	-	-*	
	**15 - β-glucuronidase**	-	-	-	-	-	-*	
	**16 - α-glucosidase**	-	-	-	-	-	-*	Regarding the literature, the ***S. xiamenensis* type strain** has α-glucosidase activity
	**17 - β-glucosidase**	-	-	-	-	-	-*	Regarding the literature, the ***S. xiamenensis* type strain** has β-glucosidase activity
	**18 - N-acetyl-β-glucosaminidase**	+	-	-	+	-	+*	Characteristic variable within ***S. seohaensis*** species
	**19 - α-mannosidase**	-	-	-	-	-	-*	
	**20 - α-fucosidase**	-	-	-	-	-	-*	
**API 20NE**	**Reduction of nitrate to nitrite/nitrogen**	+	-	+	+	-	+*	Characteristic variable within ***S. seohaensis*** species
	**Indole production**	-	-	-	-	-	-*	
	**Fermentation of glucose**	-	-	-	-	-	-*	Regarding the literature, the ***S. xiamenensis* type strain** and ***S. seohaensis* CCUG60900** can perform glucose fermentation
	**Arginine Dihydrolase**	-	-	-	-	-	-*	Regarding the literature, the ***S. xiamenensis* type strain** and ***S. seohaensis* CCUG60900** have arginine dihydrolase activity
	**Urease**	-	-	-	-	-	-*	
	**Esculin**	-	-	-	(+)	-	-*	**Distinct phenotype for *S. xiamenensis* HI32664**
	**Gelatine**	+	+	+	+	-	+*	**Distinct phenotype for *S. putrefaciens* 95**
	**β-galactosidase**	-	-	-	-	-	-*	
**API 20NE Assimilation**	**Glucose**	-	-	+	-	-	+*	Characteristic variable within ***S. seohaensis*** species
	**Arabinose**	+	+	+	+	-	-*	**Distinct phenotype for *S. putrefaciens* 95 and for *S. decolorationis* JCM21555**
	**Mannose**	-	-	-	-	-	-*	
	**Mannitol**	-	-	-	-	-	-*	
	**N-Acetyl-Glucosamine**	+	+	+	+	+	**ND**	
	**Maltose**	+	+	+	+	-	+*	**Distinct phenotype for *S. putrefaciens* 95**
	**Potassium Gluconate**	-	-	-	-	-	**ND**	

	**Capric Acid**	-	-	-	-	-	**ND**	
	**Adipic Acid**	-	-	-	-	-	**ND**	
	**Malate**	+	+	+	+	+	+*	
	**Trisodium Citrate**	-	-	-	-	-	-*	
	**Phenylacetic Acid**	-	-	-	-	-	**ND**	

Supplementary Table 3. List of exclusive genes of *S. seohaensis*, compared to closely related species. The gene selected for the PCR for species identification is represented in bold

GenBank ID of prototype	Potential product function
VEE60751.1	Phosphoglyceromutase
**VEE60752.1**	**Phosphatase PhoE**
VEE60754.1	Regulatory Protein uhpC
VEE60755.1	Phosphoglycerate Transport Regulatory Protein pgtC
VEE60756.1	Phosphoglycerate Transport System Sensor Protein pgtB
VEE60753.1	Porin porB_1
QXN24416.1	Hypothetical Protein
QXN23287.1	AraC family transcriptional regulator

Supplementary Table 4: *In-silico* PCR results, using Emboss PrimerSearch software (3), allowing 10% of mismatches. The positive results are highlighted in bold and correspond to *S. seohaensis* strains.

Assembly Accession	Primer Set	Amplimer Length	Primer Set	Amplimer Length
**BC20 (GCF_003129585.1)**	***recA***	**743 bp**	**VEE60752.1**	**609 bp**
**CCUG60900 (GCF_023283775.1)**	***recA***	**743 bp**	**VEE60752.1**	**609 bp**
**DSM9451 (This study)**	***recA***	**743 bp**	**VEE60752.1**	**609 bp**
GCA000015845.1	*recA*	743 bp	VEE60752.1	No amplification
GCA000017325.1	*recA*	743 bp	VEE60752.1	No amplification

GCA000018765.1	*recA*	743 bp	VEE60752.1	No amplification
GCA000021665.1	*recA*	743 bp	VEE60752.1	No amplification
GCA000146165.2	*recA*	743 bp	VEE60752.1	No amplification
GCA000147735.3	*recA*	743 bp	VEE60752.1	No amplification
GCA000178875.2	*recA*	743 bp	VEE60752.1	No amplification
GCA000179535.2	*recA*	743 bp	VEE60752.1	No amplification
GCA000215895.1	*recA*	743 bp	VEE60752.1	No amplification
GCA000231345.2	*recA*	743 bp	VEE60752.1	No amplification
GCA000485795.1	*recA*	743 bp	VEE60752.1	No amplification
GCA000615005.1	*recA*	743 bp	VEE60752.1	No amplification
GCA000712635.2	*recA*	743 bp	VEE60752.1	No amplification
GCA001591325.1	*recA*	743 bp	VEE60752.1	No amplification
GCA001620325.1	*recA*	743 bp	VEE60752.1	No amplification
GCA001723195.1	*recA*	743 bp	VEE60752.1	No amplification
GCA002074855.1	*recA*	743 bp	VEE60752.1	No amplification
**SA70 (GCA002157365.2)**	***recA***	**743 bp**	**VEE60752.1**	**609 bp**
**JAB-1 (GCA002216875.1)**	***recA***	**743 bp**	**VEE60752.1**	**609 bp**
GCA002738015.1	*recA*	743 bp	VEE60752.1	No amplification
GCA003028295.1	*recA*	743 bp	VEE60752.1	No amplification
GCA003030925.1	*recA*	743 bp	VEE60752.1	No amplification
GCA003044255.1	*recA*	743 bp	VEE60752.1	No amplification
GCA003052765.1	*recA*	743 bp	VEE60752.1	No amplification
GCA003130545.1	*recA*	743 bp	VEE60752.1	No amplification
GCA003241225.1	*recA*	743 bp	VEE60752.1	No amplification
GCA003315425.1	*recA*	743 bp	VEE60752.1	No amplification
GCA004354305.1	*recA*	743 bp	VEE60752.1	No amplification
GCA007595225.1	*recA*	743 bp	VEE60752.1	No amplification
GCA007595265.1	*recA*	743 bp	VEE60752.1	No amplification
GCA007595275.1	*recA*	743 bp	VEE60752.1	No amplification
GCA007595285.1	*recA*	743 bp	VEE60752.1	No amplification
GCA007595335.1	*recA*	743 bp	VEE60752.1	No amplification
GCA007636465.1	*recA*	743 bp	VEE60752.1	No amplification
GCA007923045.4	*recA*	743 bp	VEE60752.1	No amplification
GCA009730575.1	*recA*	743 bp	VEE60752.1	No amplification
GCA012490345.1	*recA*	743 bp	VEE60752.1	No amplification
GCA012490465.1	*recA*	743 bp	VEE60752.1	No amplification
GCA012490605.1	*recA*	743 bp	VEE60752.1	No amplification
GCA012584455.1	*recA*	743 bp	VEE60752.1	No amplification
GCA013267215.1	*recA*	743 bp	VEE60752.1	No amplification
GCA014647135.1	*recA*	480 bp	VEE60752.1	No amplification
GCA014647555.1	*recA*	474 bp	VEE60752.1	No amplification
GCA016406305.1	*recA*	743 bp	VEE60752.1	No amplification

GCA016406325.1	*recA*	743 bp	VEE60752.1	No amplification
GCA017068195.1	*recA*	743 bp	VEE60752.1	No amplification
GCA019266125.1	*recA*	743 bp	VEE60752.1	No amplification
GCA019266205.1	*recA*	743 bp	VEE60752.1	No amplification
GCA019457475.1	*recA*	743 bp	VEE60752.1	No amplification
GCA019457635.1	*recA*	743 bp	VEE60752.1	No amplification
GCA019599085.1	*recA*	743 bp	VEE60752.1	No amplification
GCA019599125.1	*recA*	743 bp	VEE60752.1	No amplification
GCA019655235.1	*recA*	743 bp	VEE60752.1	No amplification
GCA019655335.1	*recA*	743 bp	VEE60752.1	No amplification
GCA019655375.1	*recA*	743 bp	VEE60752.1	No amplification
GCA019973655.1	*recA*	743 bp	VEE60752.1	No amplification
GCA020216695.1	*recA*	743 bp	VEE60752.1	No amplification
GCA021209305.1	*recA*	743 bp	VEE60752.1	No amplification
GCA022321555.1	*recA*	743 bp	VEE60752.1	No amplification
GCA022453805.1	*recA*	743 bp	VEE60752.1	No amplification
GCA022662275.1	*recA*	743 bp	VEE60752.1	No amplification
GCA023283485.1	*recA*	743 bp	VEE60752.1	No amplification
GCA023283585.1	*recA*	743 bp	VEE60752.1	No amplification
GCA023283665.1	*recA*	743 bp	VEE60752.1	No amplification
GCA023283765.1	*recA*	743 bp	VEE60752.1	No amplification
GCA024655725.1	*recA*	743 bp	VEE60752.1	No amplification
GCA024803605.1	*recA*	743 bp	VEE60752.1	No amplification
GCA024971755.1	*recA*	743 bp	VEE60752.1	No amplification
GCA024972795.1	*recA*	743 bp	VEE60752.1	No amplification
GCA024998875.1	*recA*	743 bp	VEE60752.1	No amplification
GCA024998885.1	*recA*	743 bp	VEE60752.1	No amplification
GCA024998925.1	*recA*	743 bp	VEE60752.1	No amplification
GCA024998945.1	*recA*	743 bp	VEE60752.1	No amplification
GCA024998965.1	*recA*	743 bp	VEE60752.1	No amplification
GCA024998985.1	*recA*	743 bp	VEE60752.1	No amplification
GCA024998995.1	*recA*	743 bp	VEE60752.1	No amplification
GCA024999025.1	*recA*	743 bp	VEE60752.1	No amplification
GCA024999045.1	*recA*	743 bp	VEE60752.1	No amplification
GCA024999065.1	*recA*	743 bp	VEE60752.1	No amplification
GCA024999075.1	*recA*	743 bp	VEE60752.1	No amplification
GCA024999105.1	*recA*	743 bp	VEE60752.1	No amplification
GCA024999125.1	*recA*	743 bp	VEE60752.1	No amplification
GCA024999145.1	*recA*	743 bp	VEE60752.1	No amplification
GCA024999165.1	*recA*	743 bp	VEE60752.1	No amplification
GCA024999175.1	*recA*	743 bp	VEE60752.1	No amplification
GCA024999205.1	*recA*	743 bp	VEE60752.1	No amplification
GCA024999215.1	*recA*	743 bp	VEE60752.1	No amplification

GCA024999245.1	*recA*	743 bp	VEE60752.1	No amplification
GCA024999325.1	*recA*	743 bp	VEE60752.1	No amplification
GCA024999355.1	*recA*	743 bp	VEE60752.1	No amplification
GCA024999375.1	*recA*	743 bp	VEE60752.1	No amplification
GCA024999425.1	*recA*	743 bp	VEE60752.1	No amplification
GCA024999505.1	*recA*	743 bp	VEE60752.1	No amplification
GCA024999515.1	*recA*	743 bp	VEE60752.1	No amplification
GCA024999565.1	*recA*	743 bp	VEE60752.1	No amplification
GCA024999605.1	*recA*	743 bp	VEE60752.1	No amplification
GCA024999685.1	*recA*	743 bp	VEE60752.1	No amplification
GCA024999695.1	*recA*	743 bp	VEE60752.1	No amplification
GCA024999715.1	*recA*	743 bp	VEE60752.1	No amplification
GCA024999765.1	*recA*	743 bp	VEE60752.1	No amplification
GCA024999785.1	*recA*	743 bp	VEE60752.1	No amplification
GCA025370265.1	*recA*	743 bp	VEE60752.1	No amplification
GCA025397475.1	*recA*	743 bp	VEE60752.1	No amplification
GCA025397555.1	*recA*	743 bp	VEE60752.1	No amplification
GCA025397565.1	*recA*	743 bp	VEE60752.1	No amplification
GCA025397615.1	*recA*	743 bp	VEE60752.1	No amplification
GCA025397625.1	*recA*	743 bp	VEE60752.1	No amplification
GCA025397935.1	*recA*	743 bp	VEE60752.1	No amplification
GCA025402875.1	*recA*	743 bp	VEE60752.1	No amplification
GCA025653605.1	*recA*	743 bp	VEE60752.1	No amplification
GCA025653895.1	*recA*	743 bp	VEE60752.1	No amplification
GCA025919655.1	*recA*	743 bp	VEE60752.1	No amplification
GCA025998315.1	*recA*	743 bp	VEE60752.1	No amplification
GCA026012575.1	*recA*	743 bp	VEE60752.1	No amplification
GCA029719425.1	*recA*	743 bp	VEE60752.1	No amplification
GCA029839545.1	*recA*	743 bp	VEE60752.1	No amplification
GCA029843345.1	*recA*	743 bp	VEE60752.1	No amplification
GCA030008365.1	*recA*	743 bp	VEE60752.1	No amplification
GCA030008375.1	*recA*	743 bp	VEE60752.1	No amplification
GCA030008385.1	*recA*	743 bp	VEE60752.1	No amplification
GCA030008455.1	*recA*	743 bp	VEE60752.1	No amplification
GCA030008475.1	*recA*	743 bp	VEE60752.1	No amplification
GCA030008495.1	*recA*	743 bp	VEE60752.1	No amplification
GCA030008505.1	*recA*	743 bp	VEE60752.1	No amplification
GCA030008515.1	*recA*	743 bp	VEE60752.1	No amplification
GCA030008575.1	*recA*	743 bp	VEE60752.1	No amplification
GCA030008595.1	*recA*	743 bp	VEE60752.1	No amplification
GCA030008605.1	*recA*	743 bp	VEE60752.1	No amplification
GCA030008615.1	*recA*	743 bp	VEE60752.1	No amplification
GCA030012545.1	*recA*	743 bp	VEE60752.1	No amplification

GCA030160495.1	*recA*	743 bp	VEE60752.1	No amplification
GCA030161835.1	*recA*	743 bp	VEE60752.1	No amplification
GCA030281045.1	*recA*	743 bp	VEE60752.1	No amplification
GCA030848765.1	*recA*	743 bp	VEE60752.1	No amplification
GCA031456735.1	*recA*	743 bp	VEE60752.1	No amplification
GCA031583995.1	*recA*	743 bp	VEE60752.1	No amplification
GCA033031185.1	*recA*	743 bp	VEE60752.1	No amplification
GCA033031715.1	*recA*	743 bp	VEE60752.1	No amplification
GCA033484875.1	*recA*	743 bp	VEE60752.1	No amplification
GCA033843045.1	*recA*	743 bp	VEE60752.1	No amplification
GCA036324205.1	*recA*	743 bp	VEE60752.1	No amplification
GCA036324485.1	*recA*	743 bp	VEE60752.1	No amplification
GCA036326965.1	*recA*	743 bp	VEE60752.1	No amplification
GCA036326985.1	*recA*	743 bp	VEE60752.1	No amplification
GCA036327025.1	*recA*	743 bp	VEE60752.1	No amplification
GCA037094655.1	*recA*	743 bp	VEE60752.1	No amplification
GCA038432395.1	*recA*	743 bp	VEE60752.1	No amplification
GCA038432405.1	*recA*	743 bp	VEE60752.1	No amplification
GCA038432455.1	*recA*	743 bp	VEE60752.1	No amplification
GCA038432465.1	*recA*	743 bp	VEE60752.1	No amplification
GCA038432495.1	*recA*	743 bp	VEE60752.1	No amplification
GCA038432505.1	*recA*	743 bp	VEE60752.1	No amplification
GCA038432535.1	*recA*	743 bp	VEE60752.1	No amplification
GCA038432565.1	*recA*	743 bp	VEE60752.1	No amplification
GCA038432585.1	*recA*	743 bp	VEE60752.1	No amplification
GCA038432595.1	*recA*	743 bp	VEE60752.1	No amplification
GCA038432625.1	*recA*	743 bp	VEE60752.1	No amplification
GCA038432645.1	*recA*	743 bp	VEE60752.1	No amplification
GCA038432665.1	*recA*	743 bp	VEE60752.1	No amplification
GCA038432685.1	*recA*	743 bp	VEE60752.1	No amplification
GCA038432695.1	*recA*	743 bp	VEE60752.1	No amplification
GCA038432715.1	*recA*	743 bp	VEE60752.1	No amplification
GCA038432725.1	*recA*	743 bp	VEE60752.1	No amplification
GCA038432745.1	*recA*	743 bp	VEE60752.1	No amplification
GCA038432785.1	*recA*	743 bp	VEE60752.1	No amplification
GCA038432795.1	*recA*	743 bp	VEE60752.1	No amplification
GCA038432805.1	*recA*	743 bp	VEE60752.1	No amplification
GCA038432815.1	*recA*	743 bp	VEE60752.1	No amplification
GCA038432835.1	*recA*	743 bp	VEE60752.1	No amplification
GCA038432895.1	*recA*	743 bp	VEE60752.1	No amplification
GCA038432925.1	*recA*	743 bp	VEE60752.1	No amplification
GCA039762335.1	*recA*	743 bp	VEE60752.1	No amplification
GCA039871505.1	*recA*	743 bp	VEE60752.1	No amplification

GCA900156405.1	*recA*	743 bp	VEE60752.1	No amplification
GCA900456975.1	*recA*	743 bp	VEE60752.1	No amplification
GCA900457045.1	*recA*	743 bp	VEE60752.1	No amplification
GCA900457065.1	*recA*	743 bp	VEE60752.1	No amplification
**NCTC12093 (GCA900636665.1)**	***recA***	**743 bp**	**VEE60752.1**	**609 bp**
GCA900636855.1	*recA*	743 bp	VEE60752.1	No amplification
GCA902728295.3	*recA*	743 bp	VEE60752.1	No amplification
GCA945952185.1	*recA*	743 bp	VEE60752.1	No amplification
GCA949794895.1	*recA*	743 bp	VEE60752.1	No amplification
GCA963676695.1	*recA*	743 bp	VEE60752.1	No amplification
**GD03713 (GCF_029843705.1)**	***recA***	**743 bp**	**VEE60752.1**	**609 bp**
HI32665 (This study)	*recA*	743 bp	VEE60752.1	No amplification

References:

1. Yoon J-H, Park S, Jung Y-T, Lee J-S. 2012. *Shewanella seohaensis* sp. nov., isolated from a tidal flat sediment. Antonie Van Leeuwenhoek 102:149–156.

2. Huang J, Sun B, Zhang X. 2010. *Shewanella xiamenensis* sp. nov., isolated from coastal sea sediment. Int J Syst Evol Microbiol 60:1585–1589.

3. Rice P, Longden I, Bleasby A. 2000. EMBOSS: The European Molecular Biology Open Software Suite. Trends in Genetics 16:276–277.
